# Comparison of a one-step real-time RT-PCR and a nested real-time RT-PCR for a genogroup II norovirus reveals differences in sensitivity depending upon assay design and visualization

**DOI:** 10.1371/journal.pone.0248581

**Published:** 2021-04-08

**Authors:** Clyde S. Manuel, Cassandra Suther, Matthew D. Moore, Lee-Ann Jaykus

**Affiliations:** 1 Department of Food, Nutrition, and Bioprocessing Sciences, North Carolina State University, Raleigh, NC, United States of America; 2 Department of Food Science, University of Massachusetts, Amherst, MA, United States of America; Universita degli Studi di Parma, ITALY

## Abstract

Human norovirus (NoV) is the leading cause of acute viral gastroenteritis and a major source of foodborne illness. Detection of NoV in food and environmental samples is typically performed using molecular techniques, including real-time reverse transcription polymerase chain reaction (RT-PCR) and less frequently, nested real-time PCR. In this study, we conducted a controlled comparison of two published NoV detection assays: a broadly reactive one-step real-time RT-PCR and a two-step nested real-time PCR assay. A 20% human fecal suspension containing a genogroup II human NoV was serially diluted, genome extracted, and subjected to amplification using the two assays compared via PCR Units. Additional amplicon confirmation was performed by dot blot hybridization using digoxigenin (DIG)-labeled oligonucleotide probes. Both assays displayed similar amplification standard curves/amplification efficiencies; however, the nested assay consistently detected one log_10_ lower virus. Dot blot hybridization improved the detection limit of the nested real-time PCR by one log_10_ NoV genome copies but impaired the detection limit of the one-step real-time RT-PCR by one log_10_ NoV genome copies. These results illustrate the complexities in designing and interpreting molecular techniques having a sufficient detection limit to detect low levels of viruses that might be anticipated in contaminated food and environmental samples.

## Introduction

Noroviruses (NoV) are RNA viruses found within the *Caliciviridae* family. Human noroviruses are a leading cause of acute viral gastroenteritis worldwide [1]. Symptoms of NoV infection include vomiting, diarrhea, and abdominal cramping. While unpleasant, the disease is usually self-limiting but can be quite severe in certain populations, such as the elderly and younger children. Exposure to NoV occurs by direct contact with infected individuals or contaminated surfaces, from consumption of contaminated food or water, or by exposure to aerosolized vomitus [2]. Relative to foodborne illness, human NoV are responsible for over 50% of domestically acquired food-related illnesses in the U.S., equating to over 5 million cases annually [3].

Historically, the detection of human NoV in food and environmental samples relies on molecular assays such as reverse transcription-polymerase chain reaction (RT-PCR). In contrast to clinical specimens that have very high viral loads, frequently exceeding 10^9^ RT-PCR amplifiable units/g feces [4], the concentration of NoV in naturally contaminated food and environmental samples is extremely low, making detection with ligand-based techniques (like immunoassays) difficult. Given the low infectious dose of NoV, suspected to be around 20 virus particles [5], naturally contaminated food and water samples still carry disease risk. Hence, any amplification assay used for detection in these sample matrices must have a low limit of detection (LOD) [6].

Early research demonstrated less than optimal LOD for RT-PCR based detection of viruses in food or environmental sample concentrates having low levels of contamination (defined as <10^2^−10^3^ RT-PCR amplifiable units per analyte) as determined by visualization of amplicons by agarose gel electrophoresis [7]. When followed by Southern or traditional hybridization, assay detection limits improved 10-100-fold [8]. Another way to improve an assay’s detection limit is by nested PCR, which is essentially serial amplification of a target nucleic acid sequence. In theory and in practice, nested PCR has been shown to improve the detection limit and specificity of an assay [9], but the method has been criticized because of its propensity to cause amplicon contamination in laboratory settings.

In the last decade, NoV detection has been increasingly performed through real-time PCR. Further improvements to enzyme systems now allow reverse transcription and real-time PCR to be done in a single tube (one-step real-time RT-PCR), simplifying sample manipulations, and reducing the potential for amplicon contamination. Real-time RT-PCR is a major advancement because it essentially performs both amplification and confirmation in a single tube while offering an improved sensitivity (lower LOD) compared to traditional RT-PCR [10].

It is often claimed that nested PCR approaches have superior detection limits for detecting low levels of virus contamination when compared to single amplifications. However, to our knowledge, a direct comparison of nested PCR relative to single tube real-time RT-PCR for detection of human NoV has yet to be reported. In this study, we completed a controlled comparison of two detection assays that have been widely used to screen environmental samples for the presence of NoV. The assays chosen for comparison were a one-step real-time RT-PCR [11] and a nested real-time PCR [12]. Both assays have been shown to have broad specificity across genogroup II. However, genogroup II.2 (GII.2) has recently been reemerging as the cause of outbreaks in Japan and China [13, 14]. For this purpose, a GII.2 strain, Snow Mountain, was selected as the target norovirus strain. The goal of this study was to compare the detection limits of the two assays as applied to a serially diluted GII.2 human NoV stool sample.

## Materials and methods

### Sample preparation and automated nucleic acid extraction

A fecal suspension (20%; diluted in phosphate-buffered saline solution) from a volunteer previously challenged with Snow Mountain virus (GII.2) was obtained courtesy of Dr. Christine Moe, Emory University [15–18]. The fecal suspension was subjected to a brief centrifugation step (1,200 *x g* for 2 minutes) to settle residual organic matter, with recovery of the clarified supernatant. The supernatant was 10-fold serially diluted (from 10^−1^ to 10^−8^) in diethylpyrocarbonate (DEPC) treated water. One hundred μl of each dilution was used as input for RNA extraction using the automated NucliSENS® EasyMag® system (bioMérieux, Durham, NC) as per manufacturer instructions, with reconstitution of final pellet in 40 μl of proprietary buffer. RNA extracts were stored at -80°C prior to use in amplification assays.

### Assay development

#### Real-time RT-PCR assay

All primers and probes used in this study are detailed in Table 1. Mismatches of the primers with Snow Mountain Virus are provided in Table 2, as the primers for this assay are broadly reactive and validated for reactivity with other GII genotypes. All assays were replicated four times. The one-step real-time RT-PCR targeting the viral ORF1-ORF2 junction was previously reported by Jothikumar et al. (2005). Reaction tubes were prepared in a final format containing 2 μl template RNA, 200 nM of each primer (JJV2F and COG2R), 200 nM of GII probe (RING2-TP), 1x reaction mix (Invitrogen, Carlsbad, California), and 1 μl of SuperScript III RT/ Platinum *Taq* High Fidelity Enzyme Mix (Invitrogen). The reaction mixture was then subjected to a one-step thermal cycling profile using the StepOne qPCR thermal cycler (Life Technologies, Carlsbad, California) under the following amplification conditions: (i) RT for 15 min at 50°C, (ii) 2 min at 95°C, and (iii) 45 cycles of 15 s at 94°C, 15 s at 55°C, and 30 s at 72°C.

**Table 1:** Oligonucleotide primers and probes used in this work.

Oligonucleotide	Sequence (5’-3’)	Product Length	Assay	Reference
JJV2F	CAA GAG TCA ATG TTT AGG TGG ATG AG	98bp	One Step Real-Time RT-PCR	Jothikumar et al. [11]
COG2R	TCG ACG CCA TCT TCA TTC ACA		One Step Real-Time RT-PCR	Kageyama et al. [19]
RING2-TP	FAM-TGG GAG GGC GAT CGC AAT CT-BHQ		One Step Real-Time RT-PCR	Kageyama et al. [19]
JV12Y^a^	ATA CCA CTA TGA TGC AGA YTA	66bp	Nested Real-Time PCR	Vennema et al. [20]
JV13I^a^	TCA CCA TAG AAN GAG		Nested Real-Time PCR	Vennema et al. [20]
NoroGII-Fa^b^	CYT GCA CCT CMC AAT GGA	68bp	Nested Real-Time PCR	Boxman et al. [12]
NoroGII-Fb^b^	CKT GCA CCT CRC AAT GGA		Nested Real-Time PCR	Boxman et al. [12]
NoroGII-Rb^b^	TGT RAC TTC AGA GAG YGC ACA KA		Nested Real-Time PCR	Boxman et al. [12]
NoroGIIA-p^c^	CYA TCG CCC ACT GGC TYC TCA		Nested Real-Time PCR	Boxman et al. [12]
NoroGIIB-p^c^	CCA TYR CCC ACT GGC TCC TCA		Nested Real-Time PCR	Boxman et al. [12]
NoroGIIC-p^c^	CTC CAT TGC TCA TTG GCT TCT CAC G		Nested Real-Time PCR	Boxman et al. [12]

^a^ Primers used for the initial RT-PCR of the assay reported Boxman et al. 2007

^b^ Primers used for the nested real-time PCR portion of the assay reported by Boxman et al. 2007

^c^Probe was modified to include FAM at the 5’ base and TAMRA at the 3’ base.

**Table 2:** Primer mismatches for Snow Mountain Virus used in this work.

Oligonucleotide	Sequence	Nucleotide Location ^a^
JJV2F^c^	CAG GAA CCC ATG TTC AGG TGG ATG AG	5003
COG2R	TGG GAG GGC GAT CGC AAT CT	5101
RING2-TP	TGT GAA TGA AGA TGG CGT CGA	5048
JV12Y ^b^	ATA CCA CTA TGA TGC AGA **T**TA	4279
JV13I	CTC TTT CTA TGG TGA TGA TGA	4606
NoroGII-Fa ^b^^c^	C**C**T GCA CAT C**A**C AGT GGA	4479
NoroGII-Fb ^b^^c^	CCT GCA CAT C**A**C AGT GGA	4479
NoroGII-Rb ^b^^c^	TGT **G**AC TTC AGA **T**AG TGC GCA GA	4547
NoroGIIA-p ^b^	C**C**A TCG CCC ACT GGC T**C**C TCA	4500
NoroGIIB-p ^b^	CCA T**C**G CCC ACT GGC TCC TCA	4500
NoroGIIC-p^c^	TT CCA TCG CTC ATT GGC TTC TCA CG	4498

^a^Locations are aligned with Snow Mountain Virus (GenBank AY134748.1)

^b^Bold refers to the correct corresponding match to degenerate base.

^c^Underline refers to base mismatches.

#### Nested real-time PCR assay

The nested real-time PCR assay targeting the RNA-dependent RNA polymerase region of the GII NoV genome was performed as a two-step assay as previously reported [12]. For the initial RT-PCR, reaction tubes were prepared in a final format containing 5 μl template RNA, 200 nM of each primer (JV12Y and JV13I), 1x reaction mix (Invitrogen), and 1 μl of SuperScript III RT/ Platinum Taq High Fidelity Enzyme Mix (Invitrogen). The production of cDNA and the first round of amplification was performed using the following conditions: (i) RT for 15 min at 50°C; (ii) enzyme inactivation for 2 min at 95°C; and (iii) DNA amplification for 45 cycles of 30 s at 95°C, 40 s at 37°C, and 40 s at 72°C, followed by a final extension at 72°C for 5 min. The reaction tube for the nested real-time PCR was prepared in a final format containing 2.5 μl template cDNA from the initial RT-PCR, 240 nM of each primer (NoroGII-Fa, NoroGII-Fb, NoroGII-Rb), 120 nM of each FAM-labeled oligonucleotide probe (NoroGIIA-p, NoroGIIB-P, NoroGIIC-P), 1x PCR buffer (Invitrogen), 10 mM MgCl_2_, 80 μM of each deoxynucleoside triphosphate, and 2.5 U of Platinum *Taq* DNA polymerase (Invitrogen). The real-time PCR reaction was subjected to the following thermal cycling parameters: (i) 2 min at 95°C followed by 45 cycles of 15 s at 95°C, 30 s at 52°C, and 30 s at 72°C. The reactions were completed with a final extension for 5 min at 72°C.

#### Dot-blot hybridization

The RNA corresponding to each sample was re-amplified by RT-PCR and nested PCR with omission of the FAM-labeled oligonucleotide probes that are included in real-time PCR. The amplification products were used in dot-blot hybridization as an additional means of confirming the original amplification reactions. The DIG DNA Labeling and Detection Kit (Roche Diagnostics, Branchburg, NJ) was used for hybridization, in accordance with manufacturer instructions. To prepare the probes, the original unmodified (i.e., lacking quenchers and fluorophores) oligonucleotide hybridization probes from each detection assay (Table 1) were digoxigenin (DIG) labeled. For dot blot, 5 μl of PCR product was denatured by heating at 94°C for 5 min and then applied to a nylon membrane carrying positively charged quaternary ammonium groups (Roche Diagnostics). The PCR product was bound to the membrane by UV-crosslinking. Membranes were then placed into 10 ml of ExpressHyb solution (Clontech Laboratories, Mountain View CA) containing 10 μl each of the appropriate DIG-labeled hybridization probes. Hybridization was done by overnight incubation at 55°C with continuous rotation using a Hybaid oven (ThermoFisher Scientific, Waltham MA). After hybridization, membranes were washed at room temperature for 5 min in 2X SSC/0.1% SDS followed by 15 min in 0.5X SSC/ 0.1% SDS with gentle rotation. Subsequent immunological detection was achieved by treating the membranes with anti-digoxigenin alkaline phosphatase conjugate (anti-DIG-AP) followed by 5-bromo-4-chloro-3-indolyl phosphate (BCIP) and nitroblue tetrazolium salt (NBT), which resulted in purple-blue precipitates. The dot blot hybridization confirmation was repeated a total of eight times for each dilution of each detection assay.

### Standard curves and statistical analysis

Standard curves were created by 10-fold serially diluting (from 10^−1^ to 10^−8^) fecal supernatants in diethylpyrocarbonate (DEPC) treated water and then plotting cycle threshold (Ct) values against the corresponding dilution factor, and PCR Units calculated from the standard curve. Regression analysis of the standard curves produced for each assay was performed using the PROC GLM feature in SAS 9.3 (SAS Institute, Cary, NC). A *p-*value of 0.05 was chosen as the cutoff for significant results.

## Results

**Fig 1:**
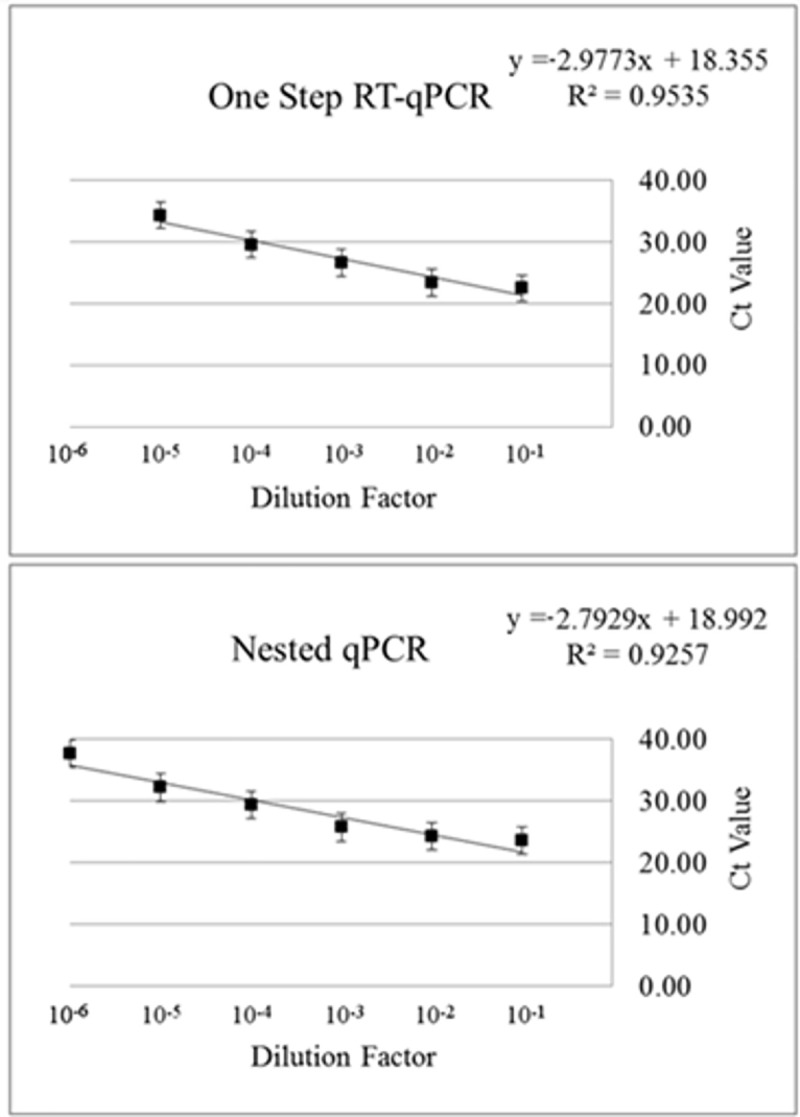
Comparison of standard curves generated using a one-step real-time RT-PCR [11] and a nested real-time PCR [12]. Each curve was generated using four replicates for each dilution sample tested. Standard error bars are shown for each curve.

Standard curves produced using the 10-fold serial dilutions of the extracted RNA displayed no significant difference (*p >* 0.05), indicating template volume had a minimal effect (when using a t-test on all dilutions) on the observed differences in the assays (S1 Fig). However, different detection limits were observed between the methods. Specifically, consistent detection (all samples from a given dilution were positive) was observed using one-step real-time RT-PCR in RNA derived from fecal suspensions diluted <10^−5^. The same consistent detection occurred by nested real-time PCR at dilutions <10^−6^ (Fig 1). Both assays also demonstrated inconsistent results (one in four replicates), with detection at 10^−6^ for the real-time RT-PCR assay, and 10^−7^ for the nested real-time PCR assay. This data is not shown in Fig 1 due to its inconsistently with the rest of the dilution replicates. However, these results suggest the nested real-time PCR had a superior LOD than the one-step real-time RT-PCR assay.

Incorporation of the dot blot hybridization step impaired the detection limit of the one-step real-time RT-PCR (Table 3). Specifically, the highest dilution at which >50% of the samples tested positive by dot blot hybridization was 10^−4^, one log_10_ worse than the FAM-labeled one-step real-time RT-PCR detection limit.

**Table 3:** Performance comparison of a one-step real-time RT-PCR and a nested real-time PCR for detection of GII.2 norovirus in fecal samples when DIG-labeled hybridization is used for amplicon confirmation^a^.

Fecal sample dilution	One-step real-time RT-PCR^b^	Nested real-time PCR^c^
10^−1^	+ (8/8)	+ (8/8)
10^−2^	+ (8/8)	+ (8/8)
10^−3^	+ (7/8)	+ (8/8)
10^−4^	+ (4/8)	+ (8/8)
10^−5^	- (0/8)	+ (8/8)
10^−6^	- (0/8)	+ (7/8)
10^−7^	- (0/8)	+ (6/8)
10^−8^	- (0/8)	- (2/8)

^a^ Samples were considered positive when the majority (i.e., 50% or greater) of replicates displayed positive signals

^b^ Source: Jothikumar et al. 2005

^c^ Source: I. L. Boxman et al. 2007

Contrary to what was observed for the one-step real-time RT-PCR, the substitution of the FAM-labeled hybridization probes with DIG-labeled hybridization probes improved the detection limit of the nested real-time PCR assay. Specifically, the highest fecal stock dilution for which >50% of samples tested positive was 10^−7^, three log_10_ better than the one-step real-time PCR detection limit (Table 3).

## Discussion

This study highlights the complexities associated with interpreting amplification results obtained when low levels of template are present. Increasingly, scientists are recognizing the need to include a confirmation step in detection assays, with cloning and sequencing being the method of choice. However, cloning and sequencing viruses in food and environmental samples that were presumptively positive by real-time RT-PCR [21] can present challenges, particularly as viruses tend to be present in the samples at low levels. While this is not a problem for human sources of norovirus, small numbers are found on environmental samples. For the purposes of this work, we have opted to compare the different assays/variables using clinical GII.2 Snow Mountain samples to reduce additional introduction of variables associated with viral inoculation/extraction in food matrices. However, based upon the results observed here, future work should focus on comparison of these assays with viruses in foods, in particular those commonly implicated in viral outbreaks. Further, it should be noted that comparison of results based on PCR Units obtained by standard curve of clinical samples rather than absolute quantification with a plasmid-based RNA standard. We chose this as we believe the co-extraction of other nucleic acids in the samples along with other components better reflects real-world samples; however, it does limit our ability to conclude absolute LOD outside of direct comparison of the different assays here.

In their original publication, Jothikumar et al. [11] compared their one-step real-time RT-PCR assay to a nested conventional RT-PCR method [22], using both assays to screen naturally contaminated shellfish for human NoV. The authors concluded that their one-step real-time RT-PCR was at least as sensitive, or similar in LOD, to the nested RT-PCR by Green et al. [22], with the added benefit of reduced time to result. However, it should be noted that the level of mismatches in the primers used (Table 2) may contribute to the difference in LOD we are observing. It should also be noted that, the nested RT-PCR by Green et al. [22] differed from that of the Boxman et al. [12] assay in several ways. For instance, the Green et al. [22] assay used random hexamers for reverse transcription, whereas the Boxman et al. [12] assay used gene-specific primers for the RT step. Random hexamers, while generally preferred for use in samples where target sequences are not known, have been shown to be associated with lower assay sensitivity and higher LOD [23]. Secondly, the early Green et al. [22] protocol used a lower annealing temperature (40°C) for the nested reactions comparative to the Boxman et al. [12] assay (52°C). In general, lower annealing temperatures can promote base-pair mismatching and the generation of non-specific amplification products, resulting in undesired increased detection limits and lower sensitivity [24]. Finally, the Boxman et al. [12] assay was reported almost a decade later than that of Green et al. [22], and it could be argued that the latter benefited from several more years of NoV sequence data, resulting in better primer/probe design. It should be noted that this comparison uses GII.2 as the only target, however, both methods were developed for the detection of multiple GII. Taken together, these differences in the comparative nested protocols between our study and that of Jothikumar et al. [11] might help explain why we observed better detection limits for the nested assay over the one-step real-time RT-PCR. Further, the Snow Mountain strain of GII.2 was used, which more commonly circulated over a few decades ago. Although this does not likely invalidate the direct comparison of the detection techniques here, future work comparing more recent GII genotypes and strains will be of value.

Another potential factor influencing the difference in both detection limits has been the fact that a slightly lower volume of template was used in the real time PCR reaction (2 μl) than the nested (5 μl) to keep reagent concentrations consistent with the methods as reported. As would be expected, the slight volumetric difference in initial template did not make a statistically significant difference (*p* > 0.05) in results (S1 Fig). It should be noted however, detection limit can be influenced by other variables between the two methods investigated as well, including integrity of primers and probes and number of amplification cycles used.

Clearly, there was difficulty in translating the one-step real-time RT-PCR to a dot blot hybridization format. While the exact mechanism for this observed impaired detection limit is not known, this observation is consistent with previous studies. For example, Houde et al. [25] reported that the substitution in a one-step real-time RT-PCR for GII NoV detection of the FAM-labeled hybridization probe with a DIG-labeled hybridization probe used in a dot blot format impaired the assay detection limit by 1000-fold. It is worth noting that the primer/probe set used by Houde et al. [25] was that initially reported by Kageyama et al. [19] and is highly like the primer/probe set we evaluated in our study. The major difference between the nested PCR reaction tubes destined for dot blot hybridization versus those used in real-time PCR was the absence of the three FAM-labeled oligonucleotide probes from the PCR reaction mix. It is well documented that assays containing high concentrations of oligonucleotides can suffer from primer dimers and/or hairpins, which can potentially out-compete template nucleic acids for reagents, particularly in the early stages of an amplification reaction [26]. In the case of the nested reactions, the absence of FAM-labeled hybridization probes in the PCR reaction mix destined for dot blot hybridization may have resulted in a decreased degree of non-specific amplification, which may explain why we observed an apparent 10-fold decrease in the nested real-time PCR assay detection limit when the DIG-labeled hybridization probes were used for amplicon confirmation. These results highlight the importance of careful PCR assay design, especially when multiple oligonucleotides are incorporated into a single reaction tube.

## Conclusion

In summary, the traditional one-step real-time RT-PCR assay had a poorer detection limit in contrast to the nested assay. A one log_10_ difference in detection limits when comparing both commonly used methods. These detection limit differences became even more disparate when dot blot hybridization was used as an alternative confirmation method for amplicon identity. One could conclude that, despite its apparent disadvantages (i.e., propensity for cross-contamination, increased time to results), well designed nested assays for NoV detection are likely better suited for situations where superior detection limits are valued over speed, as is so often the case for food and environmental samples. However, assay/primer-probe design is crucial and to date there are few nested protocols available for detection of human NoV. With limited protocols and sample complexity, challenges are likely to continue in the detection of enteric viruses in naturally contaminated food and environmental samples.

## Supporting information

S1 File Additional information. (DOCX)

S1 Fig Comparison of real-time RT-PCR using different starting volumes of RNA template. Each dilution was repeated two times for template volume tested. Standard error bars are shown for each curve. A t-test was applied to all dilutions. No significant difference was observed (*p >* 0.05).
(DOCX)
