# Supplementary material for: Comparison of a one-step real-time RT-PCR and a nested real-time RT-PCR for a genogroup II norovirus reveals differences in sensitivity depending upon assay design and visualization
Source: PLoS One. 2021 Apr 8;16(4):e0248581. doi: 10.1371/journal.pone.0248581 (PMC8031402; doi:10.1371/journal.pone.0248581)
Supplement: S1 File — (DOCX) [file pone.0248581.s001.docx]

**S1 File.**

The difference between assay efficacy when using 2μl vs 5μl of RNA template was tested to ensure that template volume was not a significant factor in the analytical sensitivity differences observed between the real-time RT-PCR and the nested PCR methods. To investigate this, the real-time RT-PCR assay was tested using 2μl vs 5μl of template and compared. It was found there was no significant difference in Ct between the two variables (*p* > 0.05). These results further indicate that both the real-time RT-PCR and the nested PCR assay methods found within this article can be compared.

**Real-time RT-PCR assay**

The assays were replicated two times with at least two reactions per sample. The one-step real-time RT-PCR targeting the viral ORF1-ORF2 junction as reported was used [11]. Reaction tubes were prepared in a final format containing either 2μl or 5μl template RNA, 200 nM of each primer (JJV2F and COG2R), 200 nM of GII probe (RING2-TP), 1x reaction mix (Invitrogen, Carlsbad, CA), and 1 μl of SuperScript III RT/ Platinum *Taq* High Fidelity Enzyme Mix (Invitrogen). The reaction mixture was then subjected to a one-step thermal cycling profile using the Bio-Rad CFX96 Touch™ Real-Time PCR thermal cycler (Bio-Rad, Berkeley, California) under the following amplification conditions: (i) RT for 15 min at 50 °C, (ii) 2 min at 95 °C, and (iii) 45 cycles of 15 s at 94 °C, 15 s at 55 °C, and 30 s at 72 °C.
