# Supplementary material for: Comparison of a one-step real-time RT-PCR and a nested real-time RT-PCR for a genogroup II norovirus reveals differences in sensitivity depending upon assay design and visualization
Source: PLoS One. 2021 Apr 8;16(4):e0248581. doi: 10.1371/journal.pone.0248581 (PMC8031402; doi:10.1371/journal.pone.0248581)
Supplement: S1 Fig — Each dilution was repeated two times for template volume tested. Standard error bars are shown for each curve. A t-test was applied to all dilutions. No significant difference was observed (p > 0.05). (DOCX) [file pone.0248581.s002.docx]

**S1 Fig. Comparison of real-time RT-PCR using different starting volumes of RNA template.** Each dilution was repeated two times for template volume tested. Standard error bars are shown for each curve. A t-test was applied to all dilutions. No significant difference was observed (*p >* 0.05).
